# Highly sensitive and selective detection of dopamine with boron and sulfur co-doped graphene quantum dots

**DOI:** 10.1038/s41598-022-13016-4

**Published:** 2022-05-31

**Authors:** Manisha Chatterjee, Prathul Nath, Sachin Kadian, Anshu Kumar, Vishal Kumar, Partha Roy, Gaurav Manik, Soumitra Satapathi

**Affiliations:** 1 Department of Biotechnology, Indian Institute of Technology Roorkee, Roorkee, Haridwar, Uttarakhand 247667 India; 2 Department of Physics, Indian Institute of Technology Roorkee, Roorkee, Haridwar, Uttarakhand 247667 India; 3 Department of Polymer and Process Engineering, Indian Institute of Technology Roorkee, Roorkee, Haridwar, Uttarakhand 247667 India

**Keywords:** Nanoscale materials, Biomedical engineering

## Abstract

In this work, we report, the synthesis of Boron and Sulfur co-doped graphene quantum dots (BS-GQDs) and its applicability as a label-free fluorescence sensing probe for the highly sensitive and selective detection of dopamine (DA). Upon addition of DA, the fluorescence intensity of BS-GQDs were effectively quenched over a wide concentration range of DA (0–340 μM) with an ultra-low detection limit of 3.6 μM. The quenching mechanism involved photoinduced electron transfer process from BS-GQDs to dopamine-quinone, produced by the oxidization of DA under alkaline conditions. The proposed sensing mechanism was probed using a detailed study of UV–Vis absorbance, steady state and time resolved fluorescence spectroscopy. The high selectivity of the fluorescent sensor towards DA is established. Our study opens up the possibility of designing a low-cost biosensor which will be suitable for detecting DA in real samples.

## Introduction

Dopamine (DA) is a well-known catecholamine that acts as a neurotransmitter within the brain and nervous system. It is found to be involved in many biological processes inside human body directly related to emotions, perception, etc. Abnormal DA concentration in biological fluids is directly linked to the detection of several diseases such as schizophrenia, anorexia and Parkinson’s disease^1,2^. As most of these diseases cannot be completely cured, medications can significantly improve the prognosis and after effects if any, can be detected at an early stage. In this regard, it is highly desirable to have both sensitive and selective detection capabilities to measure DA levels in human body for the detection of such diseases as well as for monitoring the patients already diagnosed with such conditions.

This has ignited significant interest and studies focusing on the development of analytical methods and assays for the sensitive detection of DA. Techniques involving electrochemistry^3–6^, high-performance liquid chromatography (HPLC)^7,8^, colorimetry^9–11^, capillary electrophoresis^12^ and fluorescent spectroscopy^13–18^ are applied for measuring DA concentration levels conventionally. Even though remarkable progress has been made to detect DA levels, these methods are still having limitations. Major limitations of methods like electrochemistry, colorimetry and capillary electrophoresis are low sensitivity, selectivity, bulkiness, interferences from other biomolecules, etc., which limits the development of highly efficient DA sensor. These limitations further developed more interest in fluorescence-based measurement strategies due to the simplicity, high sensitivity and efficiency compared to other strategies.

In recent years, many fluorescent chemosensors, especially quantum dots and nanoparticles, have been effectively employed for the sensitive detection of DA^19–23^. Recently, Graphene quantum dots (GQDs), zero-dimensional material, are becoming highly popular in the field of fluorescence sensing, especially due to high photostability towards photobleaching, biocompatibility and lower toxicity^24^. These unique photophysical properties of GQDs make them a potential fluorescent probe. Previously, GQDs were synthesized using “top-down” or “bottom-up” approaches like other nanomaterials^25^. For the top-down approach, cheap carbon materials can be applied as starting materials like carbon nanotubes, graphene, fullerenes etc^26–29^. But the extensive synthesis procedure, lack of control over experimental parameters, low yield, broad size distribution, etc. are some of the disadvantages^30^. The bottom-up approach utilizes smaller molecules as starting materials^31,32^ providing a more controllable strategy with more control over the optical properties, high yield and good carbonation compared to top-down approach^33^.

However, the low quantum yield of GQDs remained a constraint for fluorometric biosensing application^34^. Previous reports suggest that heteroatom doping is an effective approach for tuning the intrinsic properties of carbon nanomaterials. Many dopants in GQDs such as nitrogen^35,36^, sulfur^37–39^ and boron^40^ have been extensively studied, showing drastic alteration in photophysical properties of GQDs. Zhang et al. showed that GQDs doped with boron atoms could alter the optical properties by providing more active sites. These active sites are exploited in detecting glucose with high sensitivity and selectivity based on abnormal aggregation induced photoluminescence enhancement^41^. On the other hand, Chen et al. used nitrogen-doped GQDs for sensitive and fast detection of DA through fluorescence quenching^42^. Some reports also showed nitrogen-sulfur co-doped GQDs as an efficient fluorescent sensing probe for DA^43^, ascorbic acid^44^, mercury ions^45^, iodide and mercuric ions^46^. These reports show that single dopant GQDs have been extensively employed for rapid sensitive detection of DA but there is an open scope to examine the sensing possibility of co-dopant GQDs towards DA.

**Figure 1:**
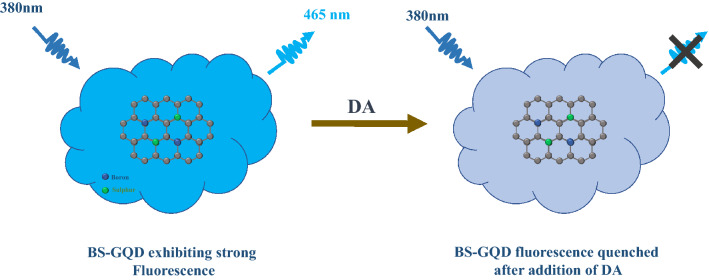
Schematic illustration of the sensing mechanism of Dopamine using BS-GQDs.

Here, we report the synthesis of boron and sulfur co-doped Graphene quantum dots (BS-GQDs) by using a simple bottom-up approach for highly sensitive and selective detection of DA. To the best of our knowledge, boron and sulfur co-doped GQDs are not reported till now and thereby in this work, we report the synthesis of BS-GQDs through one step pyrolysis and their sensory response towards dopamine. The photoluminescence of BS-GQDs showed a sharp quenching response upon addition of DA (Fig. 1). The quenching mechanism involved photoinduced electron transfer process from BS-GQDs to dopamine-quinone, produced by the oxidization of DA under alkaline conditions. The proposed sensing mechanism was investigated using a detailed study of UV–Vis absorbance, steady state and time resolved fluorescence spectroscopy.

## Experimental section

### Material and reagents

Citric acid (CA), Boric Acid (BA) and 3-Mercaptopropionic acid (MPA) were obtained from Merck, India. Sodium hydroxide (NaOH) pellets were procured from Sigma-Aldrich. All other reagents were of analytical grade and used without any modification. Fresh Millipore water was used for all experiments, dilutions and sample preparation.

### Synthesis of boron, sulfur co-doped graphene quantum dots (BS-GQDs)

Aqueous-soluble BS-GQDs were prepared through one-step thermal pyrolysis of CA (source of carbon), BA (source of boron) and MPA (source of S) using a previously reported bottom-up approach with minor modifications^47^. In brief, 1.9 g CA, 300 μL MPA and 0.2 g BA were mixed and allowed to heat up for 12 min at 200 °C. The color transformation of melted liquid from transparent to dark red during steady heat indicates the synthesis of BS-GQDs. Further, the obtained liquid was immediately poured dropwise into a freshly prepared NaOH solution (50 mL, 10 mg mL^−1^) and allowed to stir for the next 15 min. After this, the pH of the obtained solution was maintained at 7.0 and subjected to further filtration process. For purification, the as-obtained neutralized solution was first filtered through a 0.22 μm syringe filter and then subjected to dialysis using a 3 kDa dialysis bag. The resultant solution was kept in the refrigerator until further characterizations.

### Measurements and characterization

The structural properties and particle size of boron and sulfur doped Graphene quantum dots (BS-GQDs) were characterized by X-Ray Diffraction (XRD) (Rigaku smart studio X-ray diffractometer (XRD)) equipped with Cu–Kα radiation, λ = 1.5418 Å), Transmission electron microscopy (TEM) (FEI Tecnai G2 20 S-Twin, operated at 200 kV), Fourier Transform InfraRed spectroscopy (FTIR) (Carry 630, Agilent Technolgies), Quantum yield measurement (Edinburgh instruments FLS 980) and X-Ray Photoelectron Spectroscopy (XPS) (PHI 5000 Versa Probe III). For optical properties, UV–Vis absorbance measurements were carried out using Carry Win UV–Vis spectrophotometer (Agilent Technologies, United States). Fluorescence measurements were carried out using RF-6000 spectrofluorometer (Shimadzu, Japan). Time resolved fluorescence spectra were recorded with time correlated single photon counting (TCSPC) of Horiba Jobin Yvon, (Fluorocube, λ_ex_ = 375 nm, with TBX-04D photomultiplier).

### Assay

Synthesized BS-GQDs solution (200 μl) and varied concentrations of DA were added in PBS buffer solution (pH 8, 4 ml). The mixture was thoroughly mixed at room temperature. After which, the mixture is taken for recording the fluorescence measurements at an excitation wavelength of 380 nm and emission was recorded at 465 nm.

## Results and discussions

### Structural and optical characterization of BS- GQDs

**Figure 2:**
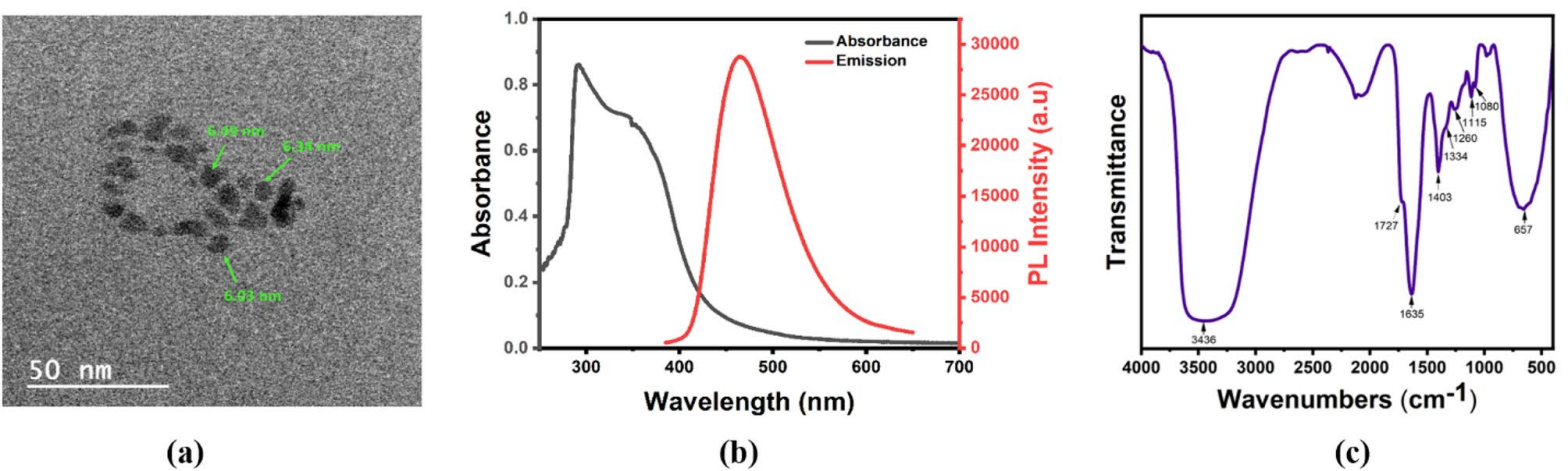
(**a**) Transmission Electron Microscope (TEM) image showing size, (**b**) Ultra Violet-Visible (UV–Vis) absorbance and (**c**) fluorescence spectra of as-synthesized Boron-Sulfur Graphene Quantum Dots (BS-GQDs).

The synthesized BS-GQDs were characterized to confirm the structural and optical properties before using it as fluorescent probe for the detection of dopamine. X-Ray diffraction pattern of BS-GQD with a broad peak (0 0 2) positioned around 20.8° conforming the formation of graphite like structure as reported before^48,49^ (Figure S1). The broad peak of the XRD pattern indicates smaller size of GQDs. The structural properties of BS-GQDs were carried out using Transmission Electron Microscopy (TEM). Figure 2a shows the TEM image with the diameter of the BS-GQDs in the range of 6 nm. The UV–Vis absorbance and fluorescence spectra of the aqueous BS-GQDs solution (Fig. 2b) showed absorbance and emission maxima at 367 nm and 465 nm, respectively. The exhibited absorbance band centered around 360 nm corresponds to n-π* transition of C=O. The excitation dependent fluorescence spectra of BS-GQDs exhibit no spectral shift with a strong fluorescence at 380 nm excitation wavelength. The absolute fluorescence quantum yield of the as synthesized BS-GQDs were found to be 19.8% which is an improvement compared to un-doped GQDs synthesized by the same approach^47^.

FTIR spectroscopy was used to investigate the presence of chemical bonding and functional groups in BS-GQDs. Figure 2c shows the FTIR spectrum with a broad intense peak due to the O–H stretching vibration centered around 3436 cm^−1^ indicating the presence of hydroxyl group and further the hydrophilic nature of the BS-GQDs^50^. The absorption bands around 1635 cm^−1^ and 1727 cm^−1^ indicates the presence of carboxylic groups^50,51^ and a minor peak around 1334 cm^−1^ indicates C–OH stretching in GQDs^52^. At 1403 cm^−1^, peaks corresponding to stretching vibration of C–H bond^53^ and B-O asymmetric stretching vibrations can be found^54^. Additionally, absorption peaks around 657 cm^−1^ are attributed to O–B–O bonds^55^ along with C–S stretching vibrations^56^ in the same region. The narrow peak around 1115 cm^−1^ is found to be the stretching vibration of C–B bond^54^ which along with other peaks proves the successful doping of Boron into the GQDs. The peaks at 1080 cm^−1^ attribute to C=S stretching^50^ and 1260 cm^−1^ is due to the symmetric stretching vibration of S=O^56^ also validates the doping of Sulfur in the GQDs.

**Figure 3:**
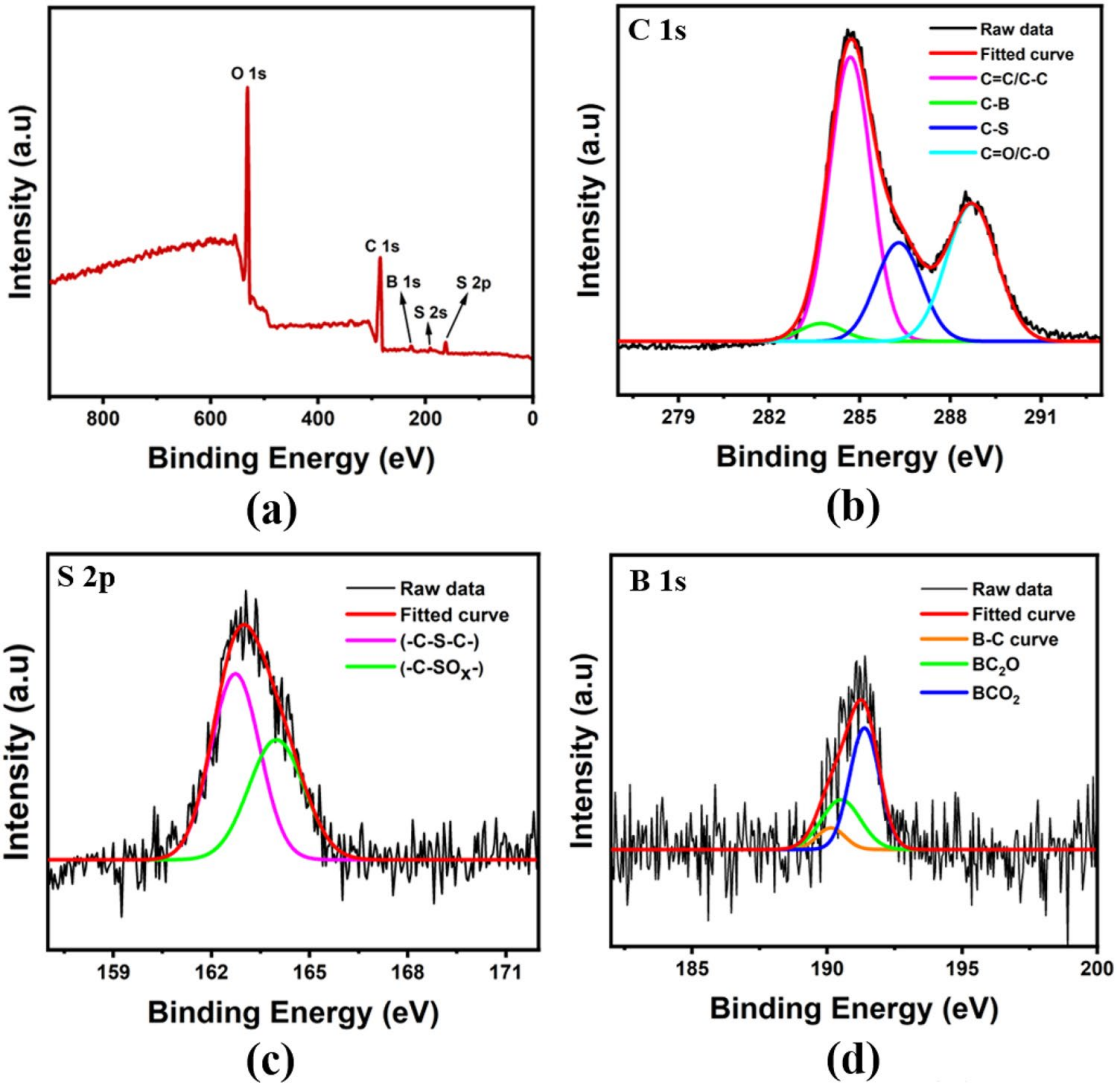
(**a**) X-Ray Photoelectron Spectroscopy (XPS) full scan survey spectrum with (**b**) high-resolution C 1s spectra, (**c**) high resolution S 2p spectra and (**d**) high-resolution B 1s spectra of BS-GQDs. The black and red lines show the raw data and fitted curve respectively whereas other colored curves show specific bonds corresponding to spectrum of each element as shown in the label.

Apart from FTIR, XPS analysis was used to study the elemental and chemical composition of the BS-GQDs. As shown in Fig. 3a, BS-GQDs comprises of five predominant peaks at binding energies around 284 eV, 531 eV, 228 eV, 191 eV and 163 eV corresponding to C 1s, O 1s, S 2s, B 1s and S 2p, respectively. The peaks at binding energies of 228 eV, 191 eV and 163 eV confirmed that S atom and B atom were successfully doped into the framework of GQDs^57,58^. The high-resolution spectra of C 1s as shown in Fig. 3b comprises of four peaks which are ascribed to C=C, C–B, C–S and C=O/C–O at binding energies of 284.7 eV, 283.7 eV, 286.3 eV and 288.7 eV, respectively^40,41,59^. The C–S peak can be seen located at lower binding energy compared to C–O peak which is due to the lower electronegativity of sulfur compared to Oxygen^60^. The S 2p spectra at 163 eV was deconvoluted into two peaks at 162.9 eV and 164.1 eV as shown in Fig. 3c. The 1st peak at 162.9 eV is attributed to 2p 1/2 and 2p 3/2 sites of the –C–S–C– covalent bond whereas the 2nd peak at 165.47 eV corresponds to –C–SOx– bond present in BS-GQDs^50,61^. The B 1s spectrum at 191 eV was further deconvoluted into three peaks at 189.8 eV, 190.6 eV, and 191.3 eV, corresponding to the B bonding in C–B (190.1 eV), BC_2_O (190.6 eV), and BCO_2_ (191.4 eV)^58^ as shown in Fig. 3d. The existence of these peaks in XPS spectra is in good accordance with FTIR spectra, thereby, confirming the successful incorporation of Boron and sulfur doping into the GQD structure as well as the formation of GQDs.

### Optimization of assay conditions

**Figure 4:**
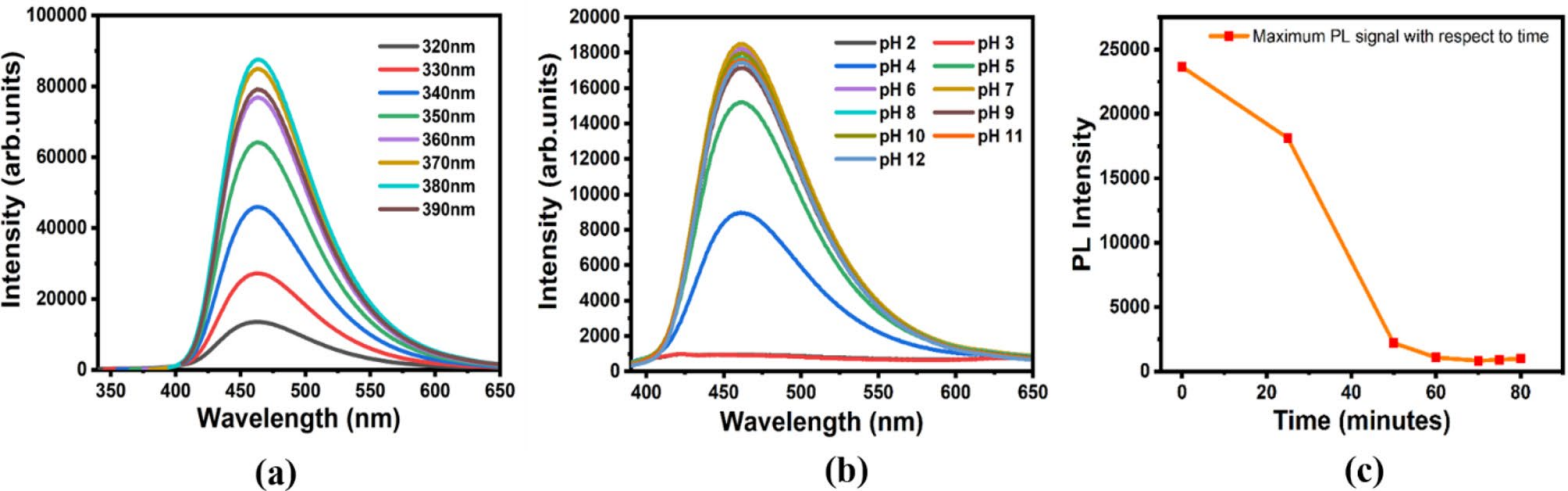
(**a**) Fluorescence response of BS-GQDs at different excitation wavelengths, (**b**) Fluorescence response of BS-GQDs at different pH values and (**c**) Time-dependent fluorescence response of BS-GQDs.

The Fluorescence emission spectra for BS-GQDs were recorded at different excitation wavelengths ranging from 300 to 390 nm showing an excitation-independent emission behavior, as shown in the Fig. 4a. This kind of emission behavior is either directly associated with the uniformity of BS-GQD size or the presence of emissive sites in the sp^2^ cluster formed^62^. pH dependent study on the buffer solution plays a major role for the sensing system as generation of dopamine-quinone^63^ as well as the fluorescence of BS-GQDs depends highly on it. For this study, we have made buffer solutions in the pH range of 2.0 to 11.0 after which the fluorescence of GQDs in each buffer is recorded as shown in Fig. 4b. It can be seen that fluorescence signals are quite high and stable in the basic region but decreased below 7.0. Basic condition of buffer favors the generation of dopamine-quinone as fluorescence quenching was comparatively high at higher pH with constant fluorescence intensity in the same region. pH optimization plays an important part for the efficient execution of the sensing strategy and it has been studied before^13^ which again showed that the optimal pH of the buffer is in the basic region. The fluorescence intensity of BS-GQDs in pH 8.0 buffer solution is found to be comparatively high, also favoring the generation of dopamine-quinone was by default used for sensing study. Figure 4c shows the optimization of incubation time which shows that the fluorescence at 465 nm of BS-GQDs continuously declined in the presence of DA over time till 80 min after which it started declining slowly.

### Detection of dopamine (DA) using BS-GQDs

**Figure 5:**
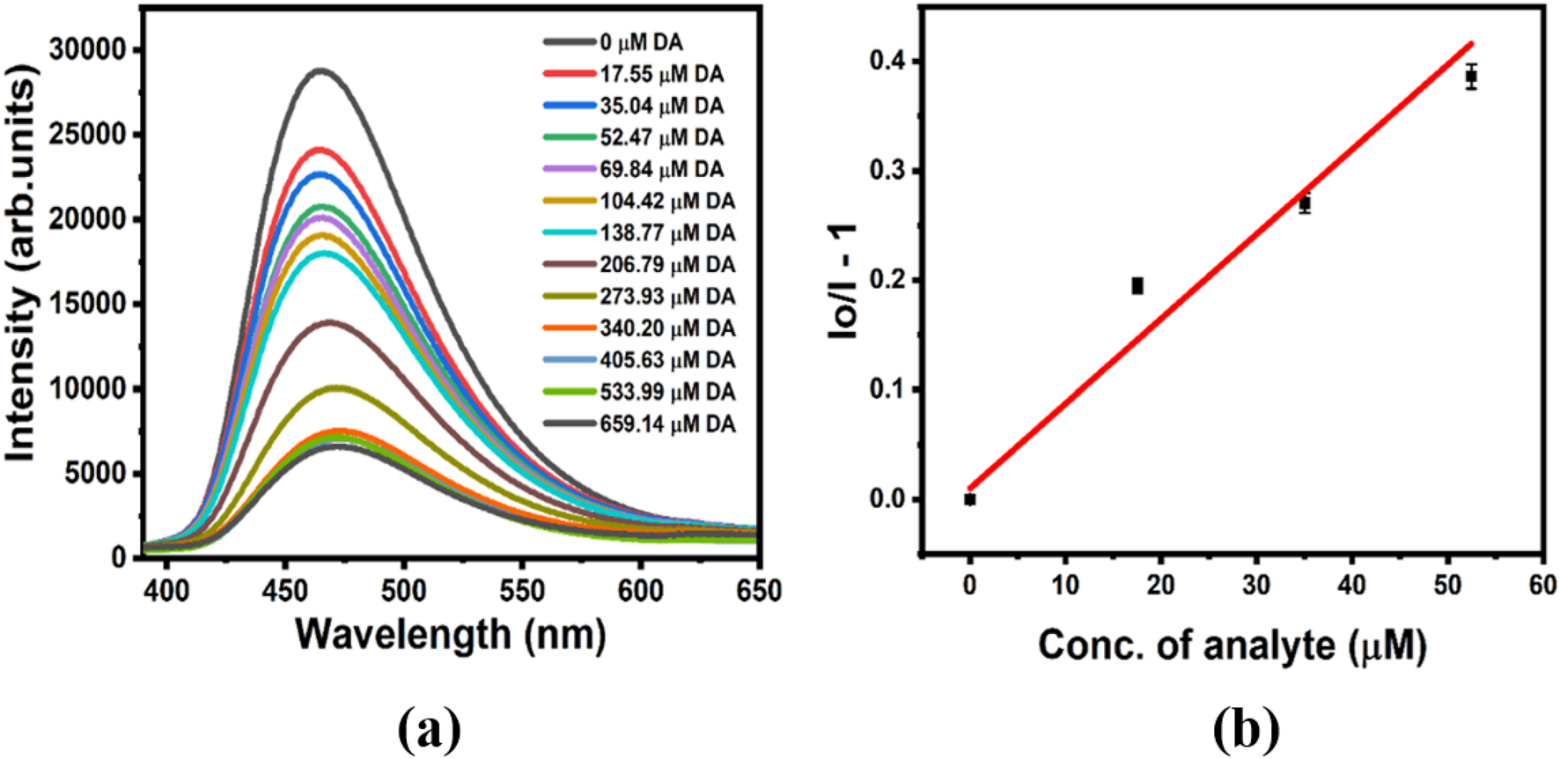
(**a**) Fluorescence response of the BS-GQDs with the addition of increasing concentration of DA and (**b**) the stern–volmer plot for the fluorescence quenching of BS-GQDs with the concentration of DA.

The sensing study using the developed fluorescence-based system for the sensitive detection of DA after optimization of the assay was carried out. The fluorescence intensity of BS-GQDs quenched linearly with the gradually increasing concentration of DA (Fig. 5a). Based on the fluorescence response after the addition of various concentrations of DA, the fluorescence quenching sensitivity is correlated and quantified with the value of stern–volmer constant (*K*_*SV*_), determined using the relation,

1 $$\frac{I}{{I}_{o}}=1+{\left[Q\right]K}_{sv}$$

where ${I}_{o}$ and I are the fluorescence intensity of the BS-GQDs at 465 nm in the absence and presence of the DA and [*Q*] is the concentration of DA. The estimated value of *Ksv* from the stern–volmer plot (Fig. 5b) is found to be 7.06 × 10^3^ M^−1^. The limit of detection (LOD) was found to be 3.6 μM with a wider linear concentration range of DA (0–340 μM). The method of LOD calculation is detailed in electronic supplementary information (ESI). So, the co-doped BS-GQDs based label-free fluorescent sensor system developed in this study shows promising outlook on co-doped GQDs for sensing.

### Probable quenching mechanism

**Figure 6:**
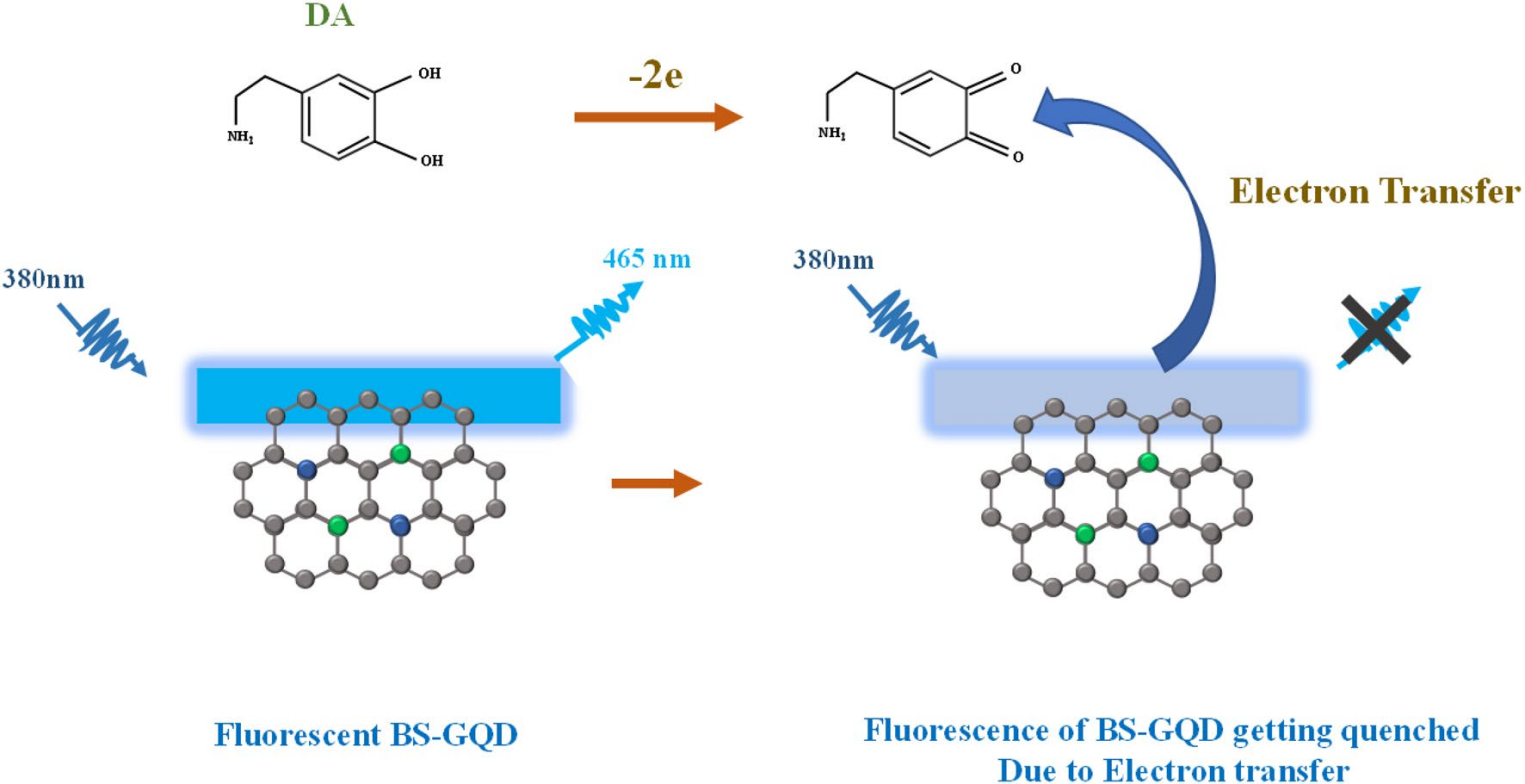
Probable mechanism involving quenching of BS-GQD fluorescence with Dopamine.

Dopamine exposed to ambient O_2_ under alkaline solution converts to dopamine-quinone which has a characteristic absorption peak around 390 nm^63^. Our experimental results (Figure S2) also confirmed the formation of dopamine-quinone by recording the absorbance spectra just before and after the incubation period for DA in basic pH buffer. It is already reported that species like dopamine-quinone acts like an electron acceptor with GQDs allowing fluorescence quenching^64–67^. Accordingly, a probable fluorescence quenching mechanism for the developed sensing system is Photoinduced Electron Transfer (PET) which enables electron transfer from BS-GQDs to dopamine quinone (Fig. 6). The carboxyl and hydroxyl groups attached on the surface of BS-GQDs effectively enables a noncovalent interaction with amine functional groups, diols and phenyl present in the DA through π-π stacking, coulombic interactions and hydrogen bonding^41^.

**Figure 7:**
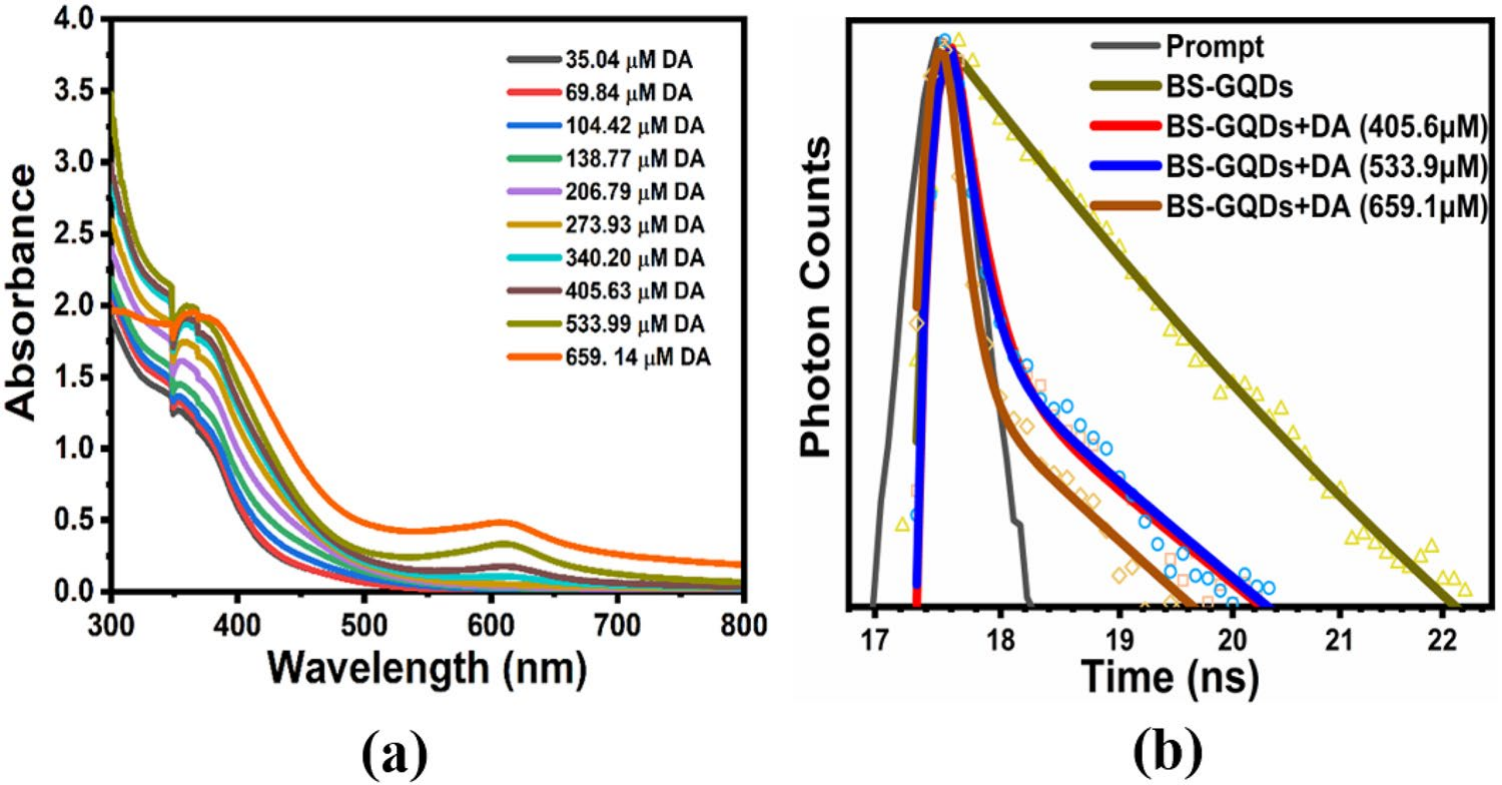
(**a**) UV–Vis absorbance spectra of BS-GQDs with successive addition of DA and (**b**) Fluorescence Lifetime decay profile of BS-GQDs without and with different concertation of DA.

To obtain an in-depth insight into the label free sensing ability of BS-GQDs for DA, UV–Vis absorbance spectra were recorded with the increasing concentration of DA. As illustrated in Fig. 7a that with the successive addition of DA with BS-GQDs, only the characteristic absorption peak of DA is intensified linearly.

To further investigate the detailed quenching mechanism, the average fluorescence lifetimes of BS-GQDs were measured in the absence and presence of different concertation of DA, using time correlated single photon counting (TCSPC) experiment (Fig. 7b). The quenching mechanism can be broadly classified into two types: static and dynamic^68^. In static quenching, a non-emissive ground state complex forms between the fluorophore and quencher. As a result, the native lifetime of the sensor system will not be affected by the addition of quencher [Q]. In dynamic quenching, the collision between the fluorophore and quencher causes electron transfer from photoexcited fluorophore to colliding quencher molecule^69^. Due to this excited state phenomena, the average lifetime of fluorophore will be decreased by the addition of quencher concentration [Q]^70^. All recorded fluorescence decay data of BS-GQDs, with and without DA, were reliably fitted to the tri-exponential decay function and tabled in Table 1. Upon addition of 405.6 μM, 533.9 μM and 659.1 μM concentration of DA, average lifetime of BS-GQDs decrease from its native average fluorescence lifetime (1.86 ns) to 0.50, 0.46 and 0.31 ns, respectively (Table 1). This significant decrease of an average lifetime of BS-GQDs with increasing concentration of DA and linear trend of Stern–Volmer plot could be attributed to the dynamic quenching process between BS-GQDs and DA^68^.

**Table 1:** Fluorescence decay parameters of BS-GQDs in the absence and presence of different concertation of DA (λ_ex_ = 375 nm, λ_em_ = 465 nm).

	A1	A2	A3	T1 (ns)	T2 (ns)	T3 (ns)	<τ> (ns)
BS-GQDs	0.42	0.01	0.57	2.28	22.12	1.03	1.86
BS-GQDs + DA (405.6 μM)	0.11	0.02	0.88	1.94	8.36	0.17	0.50
BS-GQDs + DA (533.9 μM)	0.10	0.02	0.88	1.87	8.30	0.15	0.46
BS-GQDs + DA (659.1 μM)	0.07	0.01	0.92	1.71	7.87	0.12	0.31

A comparative study of the present work with some of the reports on the detection of dopamine in solution phase using fluorescence probe is shown in Table S1 (in supplementary file). The sensory performance of the newly synthesized BS-GQDs towards dopamine detection is found to be comparable with some of the reports and offer new possibilities for further improvement in the detection strategies using GQDs.

### Photostability study of BS-GQDs

**Figure 8:**
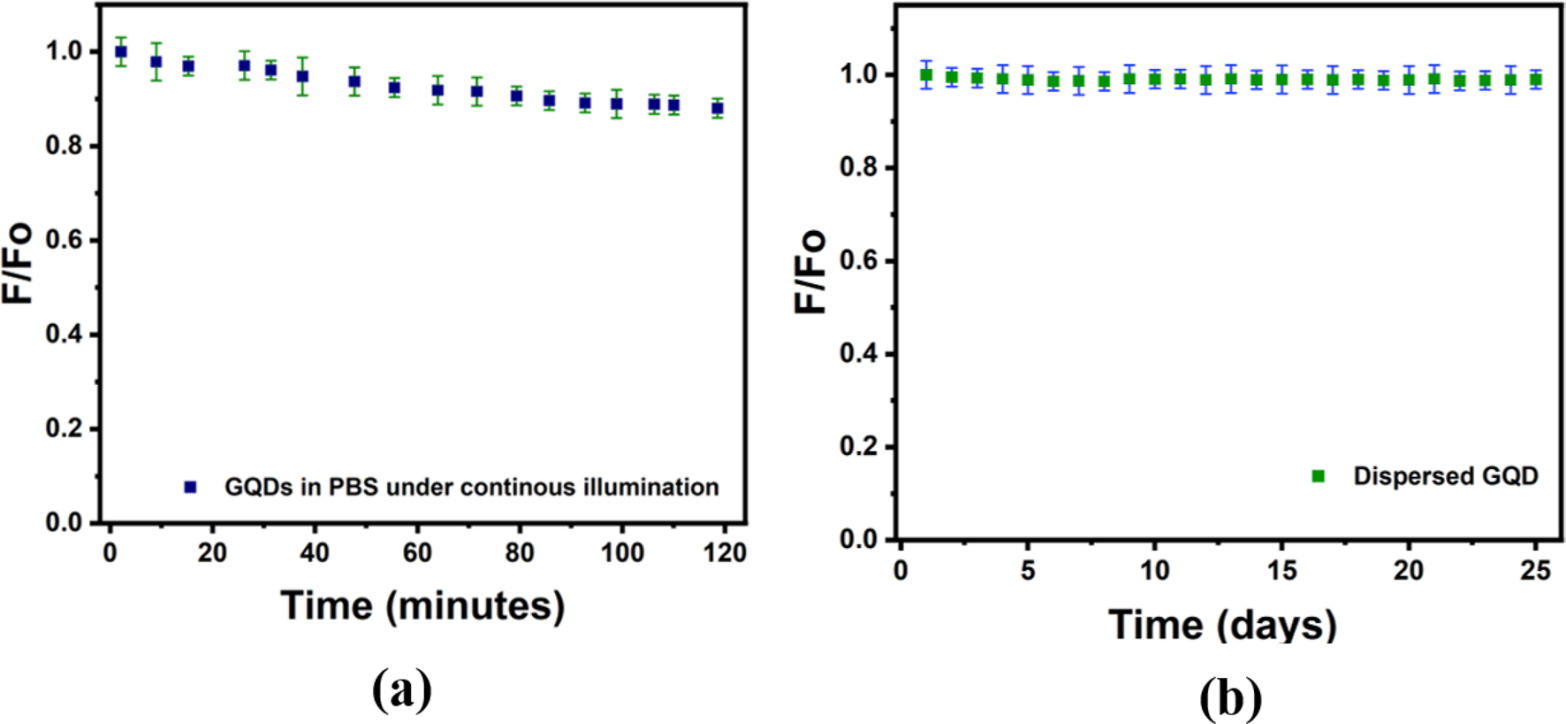
Photostability study showing the ratio of PL intensity of BS-GQDs, Fo is the initial PL intensity (at time equal to zero) and F represents PL intensity over time of BS-GQDs. (**a**) Under continuous illumination of 370 nm UV radiation (150 W Xenon lamp) at room temperature and (**b**) day wise PL spectra of GQDs stored at room temperature.

The prepared BS-GQDS are found to be highly photostable compared with other reported fluorescent Quantum dots. It can be seen from Fig. 8a that after continuous exposure of UV radiation at 370 nm (150 W Xenon lamp) inside the spectrofluorometer, around 89% of the initial fluorescence is still maintained. The prepared BS-GQDs are stable at room temperature for days maintaining strong and stable emission for 25 days showing excellent stability of the material at room temperature as can be seen in Fig. 8b.

### Selectivity for detection of DA

**Figure 9:**
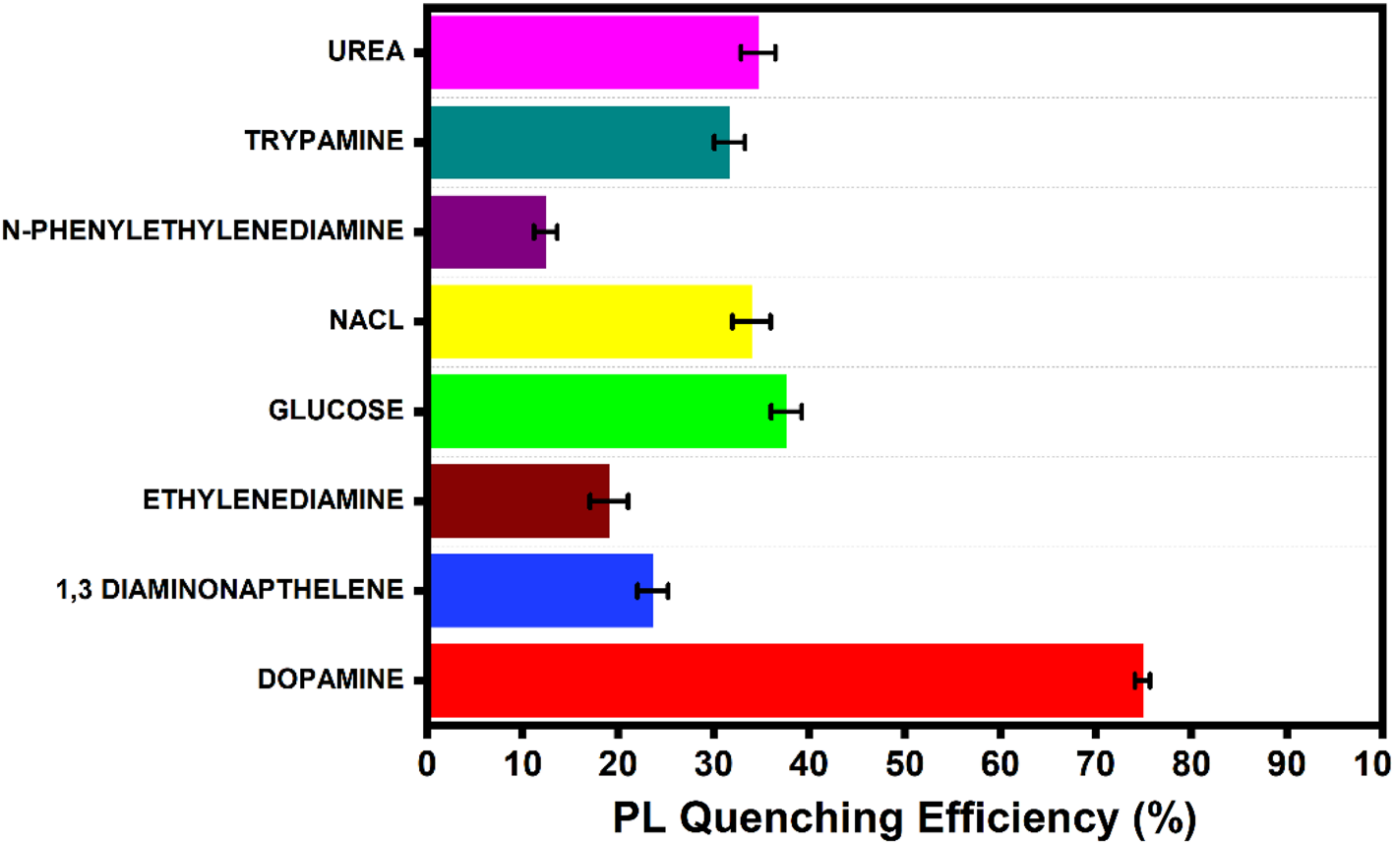
Histogram showing selectivity of the sensing system towards different species.

For the evaluation of selectivity of the proposed mechanism, several species were selected and the detection strategy was carried out for all the species. Selective detection of Dopamine relative to other species like Urea, Trypamine, N-Phenyl ethylenediamine, Sodium Chloride (NaCl), Glucose, Ethylenediamine and 1–3 Diaminonaphthalene were evaluated. The concentrations of all these species added were similar to the concentration of Dopamine added in the BS-GQD solution. As shown in the Fig. 9, no appreciable changes were observed for any other species. This confirms that the developed fluorescence sensing system is highly selective towards Dopamine.

## Conclusion

In summary, we have demonstrated fluorescent Boron and Sulfur co-doped graphene quantum dots for the efficient detection of dopamine. Dopamine effectively quenches the fluorophore’s fluorescence and charge transfer from doped quantum dots to dopamine–quinone species was proved to be responsible for fluorescence quenching. The fluorescence of the BS-GQDs was effectively quenched with the successive addition of DA. This low cost and label free method has the potential for field applications.

## Supplementary Information

Supplementary Information.
