# Supplementary material for: Highly sensitive and selective detection of dopamine with boron and sulfur co-doped graphene quantum dots
Source: Sci Rep. 2022 May 31;12:9061. doi: 10.1038/s41598-022-13016-4 (PMC9156697; doi:10.1038/s41598-022-13016-4)
Supplement: Supplementary file 1 — Supplementary Information. [file 41598_2022_13016_MOESM1_ESM.docx]

**Electronic Supplementary Information (ESI)**

**Highly Sensitive and Selective Detection of Dopamine with Boron and Sulphur co-doped Graphene Quantum Dots**

Manisha Chatterjee,^1^ Prathul Nath,^2,+^ Sachin Kadian,^3^ Anshu Kumar,^2^ Vishal Kumar,^2^ Partha Roy,^1^ Gaurav Manik,^3^ Soumitra Satapathi,^2,*^

^1^ Department of Biotechnology, Indian Institute of Technology Roorkee, Roorkee, Haridwar, Uttarakhand, 247667, India

^2^ Department of Physics, Indian Institute of Technology Roorkee, Roorkee, Haridwar, Uttarakhand, 247667, India

^3^ Department of Polymer and Process Engineering, Indian Institute of Technology Roorkee, Roorkee, Haridwar, Uttarakhand, 247667, India

**
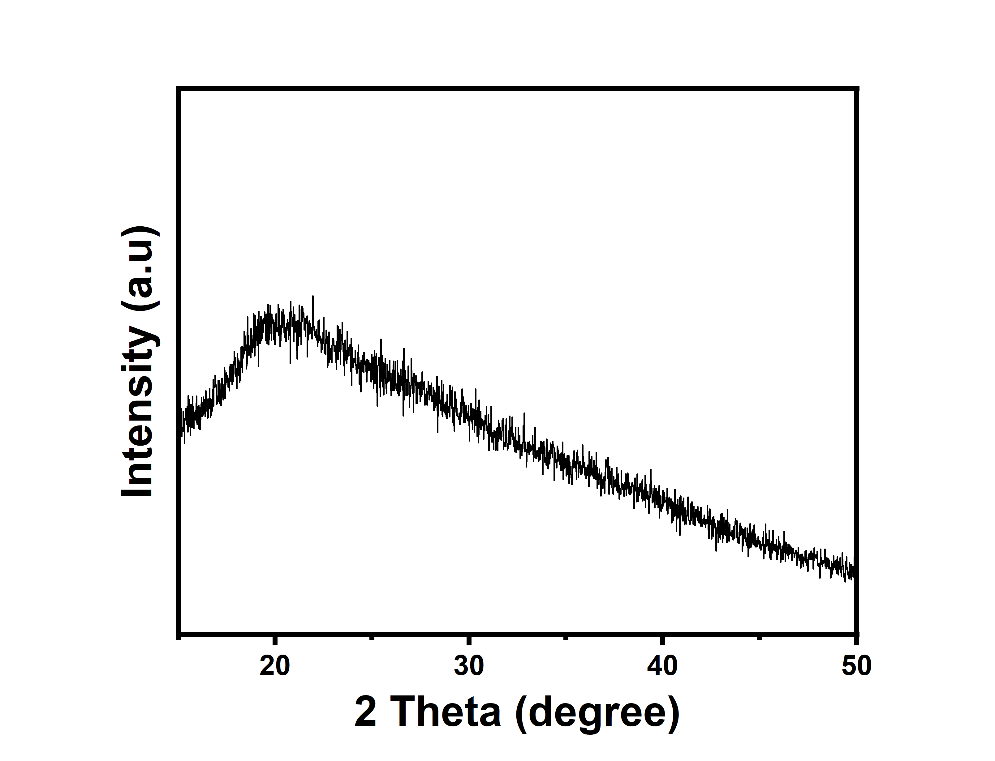
**

**Figure S1.** X-Ray diffraction pattern (XRD) of the as-synthesized Boron-Sulfur Graphene Quantum Dots (BS-GQDs)

**Figure S2.** Absorbance spectra showing formation of Dopamine quinone after 4 hours of incubation

**Calculation of Detection Limit**

The limit of detection (LOD) was estimated using the equation 3σ/K, where σ denotes the standard deviation of intensity response and K represents the slope of the calibration curve.

**Table S1.** Comparative study of some of the fluorescence-based sensors for the detection of dopamine in solution phase.

| **Sensing probe** | **Linear range (μM)** | **Limit of Detection (μM)** | **Reference** |
| --- | --- | --- | --- |
| CdTe QDs@CQDs | 10–220 mM | 0.65 mM | 1 |
| Carbon Quantum Dots | 20–100 mM | 0.2 mM | 2 |
| Silver nanoparticles | 1–100 μM | 5.39 μM | 3 |
| bCDs-gQDsrQDs | 5–50 μM | 2.7 μM | 4 |
| TGA-CdS QDs | 1.0–17.5 μM | 0.68 μM | 5 |
| Carbon Dot | 33–1250 μM | 33 μM | 6 |
| Silica coated CdTe QDs | 0.5–100 μM | 0.241 μM | 7 |
| ZnO@Cys | 26.3 - 68.5 μM | 0.791 μM | 8 |
| DA-PEI-PDA | 1–200 μM | 0.3 μM | 9 |
| Boron-Sulfur doped GQDs | 0-340 μM | 3.6 μM | This work |

**References:**

1. Xu, R., Scalco deVasconcelos, L. & Zhao, K. Computational analysis of chemomechanical behaviors of composite electrodes in Li-ion batteries. *J. Mater. Res.* **31**, 2715-2727 (2016).

2. Bharathi, D. et al. Green and cost-effective synthesis of fluorescent carbon quantum dots for dopamine detection. *J. Fluoresc.* **28**, 1-7 (2018).

3. Sivakumar, P., Priyatharshini, S. & Kumar, K. Fluorescent silver nanoparticles for sensitive and selective detection of dopamine. *Mater. Chem. Phys.* **240**, 122167 (2020).

4. Abbasi-Moayed, S., Hormozi-Nezhad, M. R. & Maaza, M. A multichannel single-well sensor array for rapid and visual discrimination of catecholamine neurotransmitters. *Sens. Actuators B Chem.* **296**, 126691 (2019).

5. Kulchat, S., Boonta, W., Todee, A., Sianglam, P. & Ngeontae, W. A fluorescent sensor based on thioglycolic acid capped cadmium sulfide quantum dots for the determination of dopamine. *Spectrochim. Acta A Mol. Biomol. Spectrosc.* **196**, 7-15 (2018).

6. Baruah, U. et al. Carbon dot based sensing of dopamine and ascorbic acid. *J. Nanopart*. **2014**, 178518-178526 (2014).

7. Ai, X. Z., Ma, Q. & Su, X. G. Nanosensor for dopamine and glutathione based on the quenching and recovery of the fluorescence of silica-coated quantum dots. *Microchim. Acta* **180**, 269-277 (2013).

8. Lin, J., Huang, B., Dai, Y. F., Wei, J. C. & Chen, Y. W. Chiral ZnO nanoparticles for detection of dopamine. *Mater. Sci. Eng. C* **93**, 739-745 (2018).

9. Wei, X., Zhang, Z. & Wang, Z. A simple dopamine detection method based on fluorescence analysis and dopamine polymerization. *Microchem. J.* **145**, 55-58 (2019).
